# Transcriptomic and RNAi-mediated antiviral response to Kamiti River virus infection in *Aedes albopictus* U4.4 cells

**DOI:** 10.1038/s41598-026-63764-w

**Published:** 2026-07-31

**Authors:** Pontus Öhlund, Marlene Cavaleiro Pinto, Anne-Lie Blomström

**Affiliations:** Department of Animal Biosciences, Swedish University of Agricultural Sciences, Box 7023, 750 07 Uppsala, Sweden

**Keywords:** Kamiti River virus, Small RNA, Transcriptome, Antiviral response, Immunology, Microbiology, Molecular biology

## Abstract

Mosquitoes transmit arboviruses that represent major global public health challenges. Increasing insecticide resistance and absence of effective antiviral therapies underscore the need for novel vector control strategies. Insect-specific viruses have emerged as candidates for biological control, however, the cellular mechanisms underlying their interactions with mosquito hosts remain poorly understood. Here, we examined the immune response of *Aedes albopictus* U4.4 cells to Kamiti River virus (KRV) infection, an insect-specific flavivirus. Cells were infected with KRV, and transcriptomic and small RNA profiles were analyzed at 24, 48 and 72 h post-infection. KRV infection induced production of virus-derived small interfering RNAs (vsiRNAs) and virus-derived PIWI-interacting RNA (vpiRNAs) from 24 to 72 h. The vsiRNAs predominantly mapped to the 3′ untranslated region of the KRV genome, whereas vpiRNAs formed distinct hotspots in regions encoding the NS1, NS3, NS4A/B and NS5 proteins. Transcriptomic analysis revealed upregulation of genes associated with the humoral immune response, including defensin, cecropin, and glutathione S-transferase, and downregulation of Toll-like receptors and ecdysone-induced transcripts at later stages of infection. These gene expression patterns suggest an early activation followed by suppression of key immune signaling pathways. Collectively, the findings indicate that KRV leads to coordinated modulation of antiviral RNAi and host transcriptional responses, consistent with a balanced, commensal-like interaction in mosquito cells.

## Introduction

Mosquitoes are globally important vectors of arthropod-borne viruses (arboviruses) that pose major threats to both veterinary and public health^1–3^. Currently, no vaccines or specific antiviral treatments are available for most arbovirus infections^4^. Control strategies therefore rely largely on reducing mosquito populations using insecticides. However, the widespread development of insecticide resistance has rendered these approaches increasingly ineffective^5^, highlighting the urgent need for novel control strategies.

One proposed approach is the release of genetically modified mosquitoes that are no longer competent vectors for arboviruses^6,7^. Achieving this requires a deeper understanding of the molecular pathways and biological processes that determine vector competence, which are closely linked to the mosquito antiviral immune response. Although mosquitoes lack adaptive immunity, several innate immune pathways, including RNA interference (RNAi), Toll, immune deficiency (Imd), and Janus kinase/signal transducer and activator of transcription (JAK/STAT), play crucial roles in the response to viral infections^8–10^. In addition, STING-like pathways have recently emerged as important components of insect antiviral immunity, contributing to the sensing of viral infection and the induction of downstream antiviral responses^11^.

RNA interference represents the primary antiviral defence in mosquitoes and comprises multiple small RNA pathways. The small interfering RNA (siRNA) pathway is the best characterised, producing virus-derived siRNAs (vsiRNAs; 21 nt in lenght) from double-stranded RNA intermediates to mediate sequence-specific viral RNA degradation. In parallel, the PIWI-interacting RNA (piRNA) pathway generates virus-derived piRNAs (vpiRNAs; 25–30 nt in lenght), particularly in persistent infections, and may contribute to antiviral defence or tolerance^8–10^. Together, these viral small RNA responses shape infection dynamics and influence vector competence.

In addition, studies have shown that the mosquito microbiome can influence the vector competence^12^. For instance, *Aedes aegypti* infected with *Wolbachia* exhibit reduced competence for dengue, Zika, and yellow fever viruses^13–15^. Similarly, insect-specific viruses (ISVs) have been shown to modulate mosquito-arbovirus interactions^16–22^. While many studies suggest that ISVs can suppress arbovirus replication, accumulating evidence indicates that their effects are context-dependent, with some ISVs facilitating subsequent arbovirus infection under certain conditions. These interactions may be mediated through mechanisms such as immune priming, competition for host resources, or superinfection exclusion, whereby prior infection with one virus limits or alters infection by a second virus^16–21,23,24^.

However, there are still knowledge gaps regarding how ISVs modulate the mosquito immune response. To explore this interaction, we conducted an in vitro study using Kamiti River virus (KRV), an insect-specific flavivrus first isolated from field-collected *Aedes macintoshi* mosquitoes in 1999^25^.

## Results

### RNAi-mediated antiviral immunity towards KRV

**Fig. 1:**
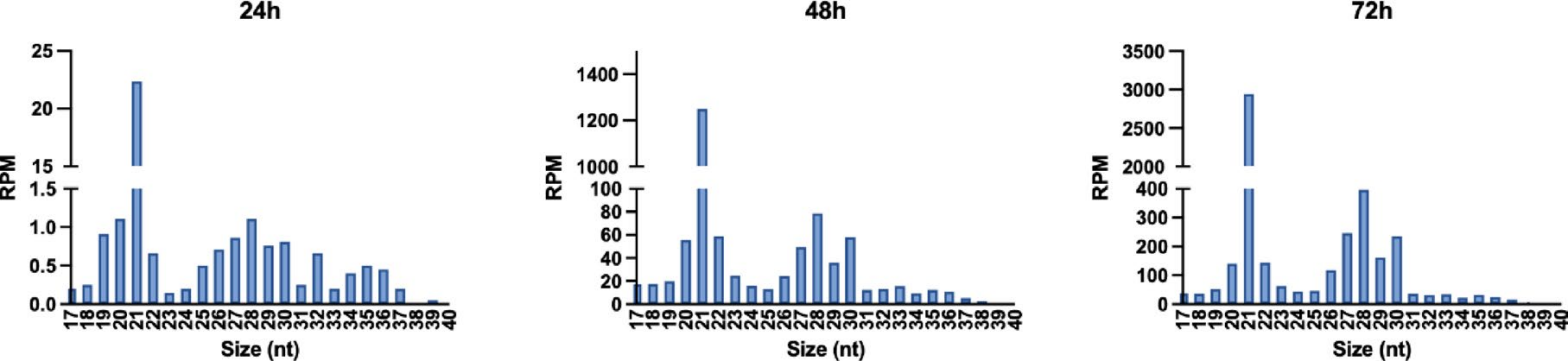
Size distribution of KRV-derived small RNAs. Small RNAs mapping to the KRV genome are shown as reads per million (RPM) normalised to total library size at 24 (reads = 19 812 507), 48 (reads = 15 959 744), and 72 hpi (16 419 790). Note that y-axis scales differ between panels.

To characterise the small RNA response to KRV infection, virus-derived small RNAs (vsRNA) were analysed across three time points (24 – 72 h). Size distribution analysis (Fig. 1) revealed that KRV-derived small RNAs were dominated by vsiRNA, as shown by the 21 nt peak, with abundance increasing markedly from 24 to 72 h post-infection (hpi). In addition, small RNAs in the 25–30 nt range also accumulated, suggesting the presence of vpiRNA.

**Fig. 2:**
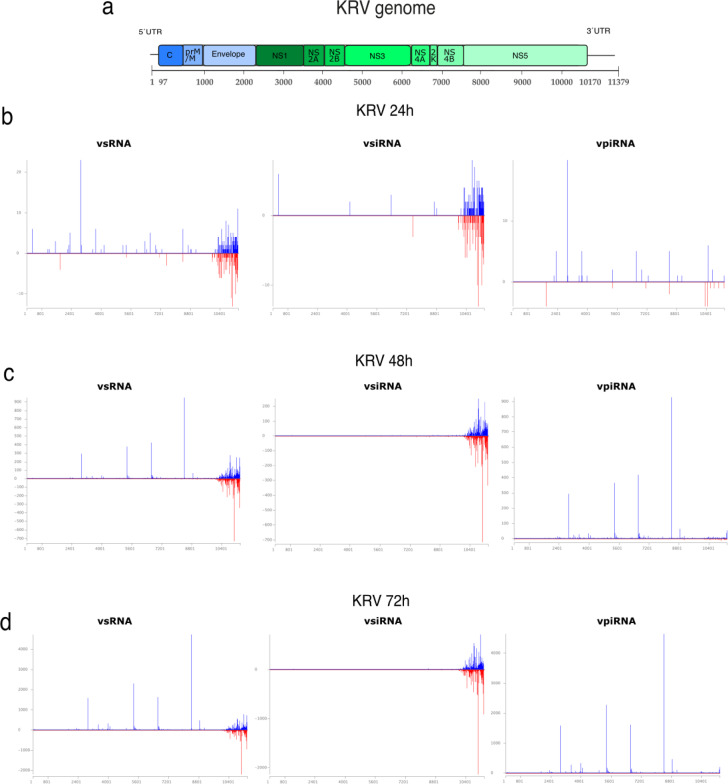
Distribution of KRV-derived small RNAs along the viral genome. (**a**) Schematic representation of the Kamiti River virus (KRV) genome, indicating structural and non-structural protein coding regions. (**b**–**d**) Distribution of virus-derived small RNAs (vsRNAs), virus-derived siRNAs (vsiRNAs; 21 nt), and virus derived piRNA-like small RNAs (25–30 nt) mapped along the KRV genome at 24 h (b), 48 h (c), and 72 h (d) post-infection. Reads mapping to the positive strand are shown in blue, and reads mapping to the negative strand are shown in red. The y-axis represents read counts, and the x-axis indicates nucleotide position along the viral genome.

The distribution of vsRNAs along the KRV genome (Fig. 2a–b) was consistent with previously reported patterns^26^. vsiRNAs (21 nt) were detected on both strands, consistent with processing of double-stranded RNA intermediates. Notably, vsiRNAs showed a pronounced enrichment toward the 3′ region of the viral genome suggesting preferential processing or accumulation of RNA derived from this region. In contrast, potential vpiRNA (25–30 nt) displayed a more uneven distribution with distinct hotspots along the viral genome. These hotspots, located in the regions coding for the NS1, NS3, NS4A/B and NS5, became more pronounced over time (Fig. 2b). Notably, these piRNA-like reads exhibited a strong strand bias, with the majority mapping to the positive strand, while comparatively few reads were detected on the negative strand.

To further investigate the nature of potential vpiRNAs, nucleotide bias at positions 1 and 10 was analysed using reads from 72 hpi, as this time point yielded the highest number of virus-derived reads and provided sufficient sequencing depth for robust analysis. Reads mapping to the positive strand exhibited a strong bias for uridine at position 1 (73%) and adenine at position 10 (67%), whereas reads mapping to the negative strand showed no pronounced nucleotide preference (26% and 25%, respectively). This asymmetry, together with the observed strand bias, is consistent with strand-specific processing characteristic of piRNA-like pathways.

### Transcriptomic analysis

**Fig. 3:**
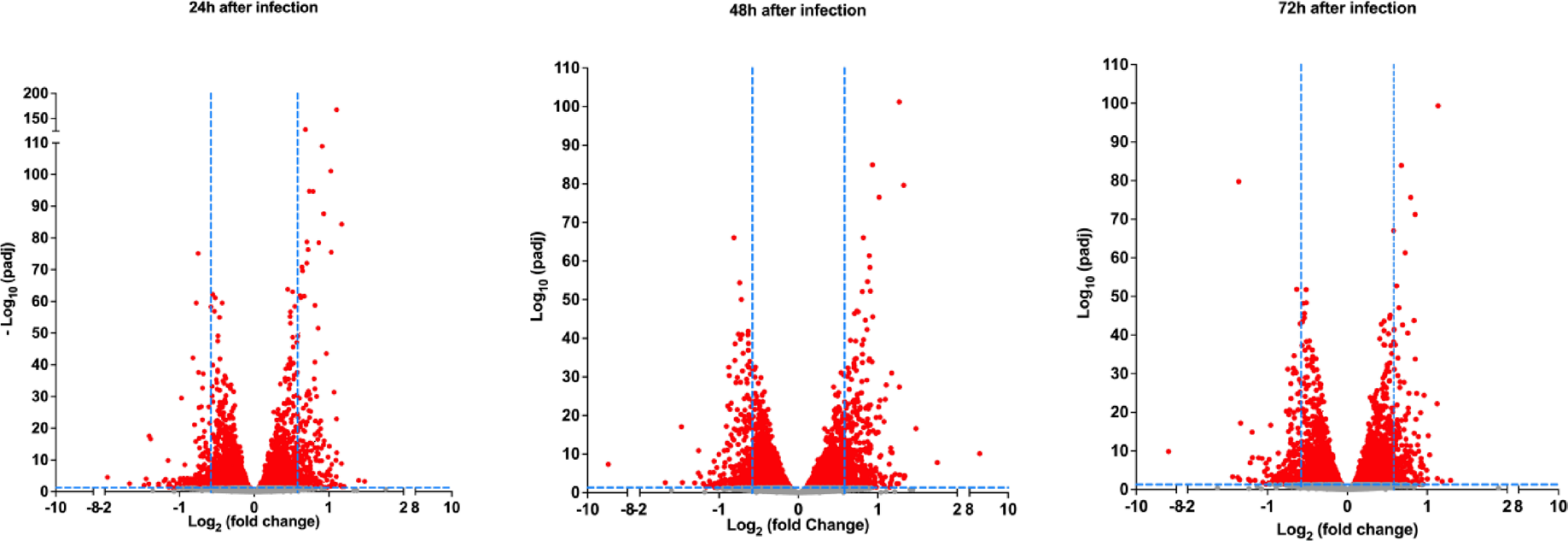
Differential expression of transcripts in *Aedes albopictus* U4.4 cells infected with KRV. Volcano plots showing the distribution of upregulated and downregulated transcripts compared to mock-infected controls at 24, 48, and 72 h post infection. Statistically significant transcripts (adjusted *p* ≤ 0.05) are shown as red dots. Vertical blue lines indicate a fold change threshold of ± 1.5, while the horizontal line denotes the adjusted p-value cutoff (padj = 0.05).

**Fig. 4:**
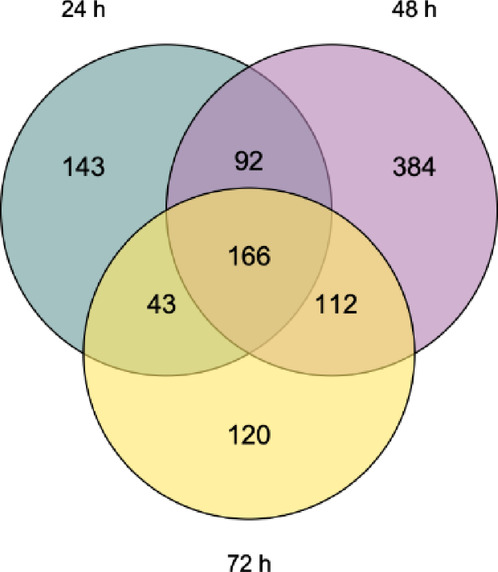
Distribution of differentially expressed transcripts shared and unique across timepoints. Venn diagram showing the overlap and unique distribution of differentially expressed transcripts (adjusted *p* ≤ 0.05; fold change ≥ 1.5) across the three time points (24, 48, and 72 h post infection).

In addition to profiling small RNAs, we analyzed mRNA sequences to identify differentially expressed (DE) transcripts involved in the mosquito immune response following KRV infection. At each time point, a substantial number of transcripts were significantly DE (adjusted *p* ≤ 0.05) compared to the mock-infected control group, ranging from 3,379 to 3,880 (Fig. 3). However, the magnitude of change was generally moderate, with only 444 (24 hpi), 754 (48 hpi), and 441 (72 hpi) of these transcripts exhibiting a fold change (FC) ≥ 1.5. At 24 hpi, 59% of DE transcripts were upregulated and 42% were downregulated. At 48 h, 63% were upregulated and 36% downregulated, while at 72 h, 54% were upregulated and 46% downregulated. Comparative analysis across time points revealed 166 DE transcripts shared among all three time points, while 143, 384, and 120 DE transcripts were unique to 24, 48, and 72 h, respectively (Fig. 4).

As noted, the fold changes were modest compared to the mock-infected control group, however, among the upregulated transcripts there was one exception and that was for the transcript encoding the succinate dehydrogenase cytochrome b small subunit (AALF005278-RA), which exhibited an exceptionally high expression (FC = 370.17) at 48 h. With the exception of AALF005278-RA, the FC values for the top 20 most upregulated transcripts ranged from 3.36 to 1.89 across time points (Table 1). A similar range (3.90–1.49) was observed for the top 20 downregulated transcripts (Table 2). However, at 48 h and 72 h post infection, one transcript (AALF003258-RA; Forkhead-associated (FHA) domain, G-patch domain, SMAD/FHA domain superfamily) stood out amongst the downregulated transcripts, with a decreased expression equal to a FC of 497.14 and 333.22, respectively (Table 2).

**Table 1:** Top 20 upregulated transcripts with the highest fold change at 24, 48, and 72 h post infection (hpi) with Kamiti River virus (KRV).

Transcript ID	Product Description [orthologue]*	Gene ontology terms*	FC
*24 h post infection*			
AALF026732-RA	u **[**phospholipase A2 hemilipin-like**]**	arachidonate transport	2.79
AALF021888-RA	unspecified product	Information not available	2.64
AALF026252-RA	**[**ribosome quality control complex subunit 2-like**]**	Information not available	2.31
AALF022023-RA	unspecified product	Information not available	2.29
AALF016193-RA	Dynein assembly factor 3, C-terminal domain; Dynein assembly factor 3, axonemal	axonemal dynein complex assembly	2.28
AALF019977-RA	Complex 1 LYR protein	Information not available	2.26
AALF017185-RA	Putative DNA-binding nuclear protein p8; Nuclear protein 1-like	DNA binding	2.25
AALF002158-RA	Yos1-like	integral component of membrane	2.25
AALF028132-RA	Succinate dehydrogenase assembly factor 3	mitochondrial respiratory chain complex II assembly	2.22
AALF018243-RA	RNA polymerase subunit RPABC4/transcription elongation factor Spt4; DNA-directed RNA polymerases I, II, and III subunit RPABC4	transcription, DNA-templated	2.15
AALF005471-RA	40S ribosomal protein S21	translation; peptide metabolic process; peptide biosynthetic process; amide biosynthetic process	2.15
AALF002626-RA	40S ribosomal protein S21	translation; peptide metabolic process; peptide biosynthetic process; amide biosynthetic process	2.15
AALF027815-RA	unspecified product	Information not available	2.14
AALF014650-RA	Cecropin	antibacterial humoral response; defense response to bacterium; immune system process; innate immune response	2.14
AALF009679-RA	unspecified product	integral component of membrane	2.12
AALF012914-RA	ATP synthase membrane subunit DAPIT	mitochondrial proton-transporting ATP synthase complex	2.10
AALF020582-RA	TCF3 fusion partner	apoptotic process	2.06
AALF011160-RA	Putative histone h3 lys4 methyltransferase complex subunit; Dpy-30 motif; Sdc1/DPY30	MLL3/4 complex; Set1C/COMPASS; methylation	2.06
AALF007837-RA	Complex 1 LYR protein	Information not available	2.05
AALF011737-RA	**[**Gag-like protein**]**	Information not available	2.04
*48 h post infection*			
AALF005278-RA	Succinate dehydrogenase cytochrome b small subunit	Information not available	370.17
AALF010775-RA	CHCH	Information not available	3.36
AALF010933-RA	UPF0708 protein C6orf162	integral component of membrane	2.79
AALF012753-RA	IPR010625	Information not available	2.53
AALF006184-RA	unspecified product	Information not available	2.52
AALF006885-RA	Intraflagellar transport protein 20	Information not available	2.51
AALF017185-RA	Putative dna-binding nuclear protein p8; Nuclear protein 1-like	DNA binding	2.51
AALF024303-RA	Nuclear receptor-binding factor 2, MIT domain; Nuclear receptor-binding factor	Autophagy	2.42
AALF014036-RA	Domain of unknown function DUF4536	integral component of membrane	2.41
AALF005471-RA	40S ribosomal protein S21	structural constituent of ribosome	2.41
AALF012074-RA	unspecified product	Information not available	2.39
AALF009378-RA	Trypsin Inhibitor-like, cysteine rich domain; Serine protease inhibitor-like superfamily	Information not available	2.36
AALF022023-RA	unspecified product	Information not available	2.33
AALF011160-RA	Putative histone h3 lys4 methyltransferase complex subunit	methyltransferase activity; transferase activity; methylation	2.27
AALF000656-RA	Cecropin-A2	antibacterial humoral response; defense response to bacterium; immune system process; innate immune response	2.27
AALF017826-RA	Cytochrome c oxidase subunit 6C; Mitochondrial cytochrome c oxidase subunit VIc/VIIs; Mitochondrial cytochrome c oxidase subunit VIc/VIIs superfamily	cytochrome-c oxidase activity; electron transport chain; proton transmembrane transport	2.26
AALF015657-RA	ubiquitin	protein binding	2.25
AALF001709-RA	Succinate dehydrogenase assembly factor 4	Information not available	2.24
AALF013073-RA	**[**CSON009053 protein**]**	integral component of membrane	2.24
AALF001425-RA	gamma-soluble nsf attachment protein (snap)	Information not available	2.23
*72 h post infection*			
AALF025937-RA	DNA recombination and repair protein Rad51-like, C-terminal; DNA recombination and repair protein RecA-like, ATP-binding domain; P-loop containing nucleoside triphosphate hydrolase	ATP binding; ATP-dependent activity, acting on DNA; DNA binding DNA repair	2.45
AALF021740-RA	Tricarboxylate/iron carrier	ion transmembrane transporter activity	2.28
AALF017185-RA	Putative dna-binding nuclear protein p8; Nuclear protein 1-like	DNA binding	2.20
AALF007289-RA	MADF domain; Transcription factor Adf-1	Information not available	2.19
AALF002626-RA	40S ribosomal protein S21	structural constituent of ribosome; translation	2.18
AALF009815-RA	Putative cpij018896 serine threonine-protein kinase; THAP-type zinc finger	DNA binding; kinase activity; nucleic acid binding; phosphorylation	2.04
AALF012753-RA	CHCH	Information not available	2.02
AALF006933-RA	Putative salivary secreted peptide	integral component of membrane	2.00
AALF011149-RA	LPS-induced tumor necrosis factor alpha factor; LITAF domain containing protein	integral component of membrane	1.99
AALF026322-RA	Sel1-like repeat	protein binding	1.95
AALF013352-RA	BLOC-1 complex, subunit 3	Information not available	1.93
AALF028074-RA	Marvel domain	integral component of membrane	1.93
AALF012682-RA	Putative neuroproteinsis	integral component of Golgi membrane; protein transport	1.92
AALF028007-RA	unspecified product	Information not available	1.91
AALF014770-RA	Transmembrane protein 42	integral component of membrane	1.90
AALF011160-RA	Putative histone h3 lys4 methyltransferase complex subunit; Dpy-30 motif; Sdc1/DPY30	methyltransferase activity; transferase activity; methylation	1.90
AALF008233-RA	unspecified product	Information not available	1.90
AALF019485-RA	Brix domain	Information not available	1.89
AALF010887-RA	Small integral membrane protein 20	integral component of membrane; mitochondrion	1.89
AALF002158-RA	Yos1-like	integral component of membrane	1.89

*Information extracted from VectorBase; FC – fold change.

**Table 2:** Top 20 downregulated transcripts with the highest fold change at 24, 48, and 72 h post infection (hpi) with Kamiti River virus (KRV).

Transcript ID	Product Description [orthologue]*	Gene ontology terms*	FC
*24 h post infection*			
AALF027399-RA	Retrotransposon; Ribonuclease H-like superfamily; Integrase zinc-binding	nucleic acid binding; DNA integration	3.90
AALF017777-RA	DM DNA-binding domain	DNA-binding transcription factor activity; metal ion binding; sequence-specific DNA binding; regulation of transcription	3.18
AALF021729-RA	RNA recognition motif domain	RNA binding; mRNA 3’-UTR binding; nucleic acid binding; translation regulator activity	2.78
AALF003314-RA	**[**girdin-like; myosin-7-like**]**	Information not available	2.73
AALF019086-RA	P-type ATPase; subfamily IV; transmembrane domain superfamily; probable phospholipid-transporting ATPase	ATP binding; magnesium ion binding; ATPase-coupled intramembrane lipid transporter activity	2.66
AALF022033-RA	Toll/interleukin-1 receptor homology (TIR) domain;	protein binding; signal transduction	2.65
AALF013096-RA	DNA binding HTH domain	DNA binding	2.62
AALF020490-RA	Exostosin-like; Exostosin-1; Exostosin, GT47 domain	glucuronosyltransferase activity; glycosyltransferase activity; heparan sulfate proteoglycan biosynthetic process;	2.43
AALF011341-RA	Rab-GTPase-TBC domain	Information not available	2.43
AALF002163-RA	matrix metalloproteinase; peptidoglycan binding-like	hydrolase activity; metalloendopeptidase activity; zinc ion binding; proteolysis	2.31
AALF010631-RA	Receptor protein-tyrosine kinase	transmembrane receptor; protein tyrosine kinase signaling pathway	2.27
AALF026882-RA	Occludin homology domain; RNA polymerase II elongation factor ELL	transcription elongation from RNA polymerase II promoter	2.23
AALF012109-RA	Reverse transcriptase domain	RNA-directed DNA polymerase activity	2.22
AALF008155-RA	Reverse transcriptase domain	RNA-dependent DNA biosynthetic process	2.21
AALF019772-RA	P-type ATPase, transmembrane domain superfamily; P-type ATPase, cytoplasmic domain N; HAD-like superfamily	ATPase-coupled intramembrane lipid transporter activity; magnesium ion binding; phospholipid transport	2.20
AALF012953-RA	unspecified product	Information not available	2.18
AALF013707-RA	Cadherin-like; Cadherin conserved site	homophilic cell adhesion via plasma membrane adhesion molecules	2.13
AALF011930-RA	RNA recognition motif domain	RNA binding; nucleic acid binding	2.12
AALF002509-RA	Cortactin-binding protein-2, N-terminal	Information not available	2.07
AALF012749-RA	K Homology domain, type 1 superfamily	RNA binding; nucleic acid binding	2.06
*48 h post infection*			
AALF003258-RA	Forkhead-associated (FHA) domain; G-patch domain; SMAD/FHA domain superfamily	nucleic acid binding; protein binding	497.14
AALF000017-RA	Unspecified product	Information not available	3.20
AALF022033-RA	Toll/interleukin-1 receptor homology (TIR)	protein binding; signal transduction	2.78
AALF021784-RA	gliotactin	integral component of membrane; membrane	2.76
AALF007077-RA	Retrotransposon, Pao; DNA/RNA polymerase superfamily	Information not available	2.48
AALF019918-RA	**[**serine/threonine-protein kinase tousled-like 2**]**	Information not available	2.40
AALF013096-RA	DNA binding HTH domain, Psq-type	DNA binding	2.39
AALF003314-RA	**[**girdin-like; myosin-7-like**]**	Information not available	2.35
AALF006782-RA	Reverse transcriptase, RNA-dependent DNA polymerase	Information not available	2.29
AALF015521-RA	DNA polymerase V/Myb-binding protein 1A; Armadillo-type fold	DNA binding; transcription factor binding; regulation of transcription	2.29
AALF021137-RA	Reverse transcriptase/retrotransposon-derived protein, RNase H-like domain; DNA/RNA polymerase superfamily	RNA-directed DNA polymerase activity; aspartic-type endopeptidase activity; nucleic acid binding	2.24
AALF005796-RA	catalytic core; Retrotransposon, Ribonuclease H-like superfamily; Integrase zinc-binding domain	nucleic acid binding; DNA integration	2.23
AALF013226-RA	catalytic core; Retrotransposon, Ribonuclease H-like superfamily; Integrase zinc-binding domain	nucleic acid binding; DNA integration	2.23
AALF025879-RA	Armadillo-type fold	Information not available	2.18
AALF013865-RA	Reverse transcriptase domain; Ribonuclease; Endonuclease/exonuclease/phosphatase	RNA–DNA hybrid ribonuclease activity; RNA-directed DNA polymerase activity; nucleic acid binding	2.16
AALF010406-RA	**[**GATA zinc finger domain-containing protein 14-like**]**	Information not available	2.15
AALF009656-RA	Rh7-like sensitivity opsin	G protein-coupled receptor activity; photoreceptor activity; response to stimulus; signal transduction	2.10
AALF006773-RA	Low-density lipoprotein (LDL) receptor class A repeat	protein binding	2.10
AALF010944-RA	Adenylyl cyclase class-3/4/guanylyl cyclase; Nucleotide cyclase	lyase activity; nucleotide binding; phosphorus-oxygen; cyclic nucleotide biosynthetic process; intracellular signal transduction	2.09
AALF003467-RA	Tuberin/Ral GTPase-activating protein subunit alpha	GTPase activator activity; positive regulation of GTPase activity	2.08
*72 h post infection*			
AALF003258-RA	Forkhead-associated (FHA) domain; G-patch domain; SMAD/FHA domain superfamily	nucleic acid binding; protein binding	333.22
AALF007077-RA	Retrotransposon, Pao; DNA/RNA polymerase superfamily	Information not available	2.70
AALF023654-RA	DNA/pantothenate metabolism flavoprotein, C-terminal; CoaB-like superfamily	Information not available	2.59
AALF011390-RA	ecdysone-induced protein	peptide-methionine (S)-S-oxide reductase activity	2.57
AALF024063-RA	Zinc finger C2H2-type	nucleic acid binding	2.54
AALF022033-RA	Toll-like receptor	protein binding; cellular response to stimulus	2.52
AALF011440-RA	unspecified product	Information not available	2.36
AALF006773-RA	[transcript variant X2]	Information not available	2.32
AALF020855-RA	**[**CNMamide**]**	Information not available	2.32
AALF013096-RA	DNA binding HTH domain, Psq-type; Homeobox-like domain superfamily	DNA binding	2.28
AALF011935-RA	**[**serine-rich adhesin for platelets, transcript variant X2**]**	Information not available	2.28
AALF008155-RA	RNA-directed DNA polymerase	RNA-directed DNA polymerase activity; RNA-dependent DNA biosynthetic process	2.23
AALF026882-RA	Occludin homology domain; RNA polymerase II elongation factor ELL, N-terminal; ELL/occludin family	transcription elongation from RNA polymerase II promoter	2.16
AALF029906-RA	Metazoan signal, recognition particle RNA	Information not available	2.10
AALF027399-RA	Integrase, catalytic core; Retrotransposon, Pao; Ribonuclease H-like superfamily; Domain of unknown function DUF5641; Integrase zinc-binding domain	nucleic acid binding; DNA integration	2.08
AALF019918-RA	**[**serine/threonine-protein kinase tousled-like 2, transcript variant X6**]**	Information not available	2.05
AALF010944-RA	Adenylyl cyclase class-3/4/guanylyl cyclase	cyclic nucleotide biosynthetic process; intracellular signal transduction	2.03
AALF014333-RA	unspecified product	Information not available	2.02
AALF007903-RA	Zinc finger, CCHC-type	nucleic acid binding; zinc ion binding	2.01
AALF012760-RA	**[**afadin-like, transcript variant X5**]**	Information not available	1.49

*Information extracted from VectorBase; FC – fold change.

Among upregulated transcripts, we identified genes associated with cellular homeostasis (e.g., succinate dehydrogenase, phospholipase A2 hemilipin-like, ribosome quality control complex subunit 2-like, ATP synthase membrane subunit DAPIT, Sdc1/DPY30, cytochrome c oxidase subunit 6C, ubiquitin and nuclear receptor-binding factor 2), immune responses (e.g., cecropin-A2, histone H3 Lys4 methyltransferase, glutathione transferase), and inflammatory processes (e.g., putative DNA-binding nuclear protein p8) (Table 1). Within the immune-related group, cecropin transcripts were detected at 24 h (AALF014650-RA, FC = 2.14) and 48 h (AALF000656-RA, FC = 2.27) post infection. Cecropins are antimicrobial peptides contributing to humoral immune responses (GO analysis, Table 1). Additionally, histone H3 Lys4 methyltransferase transcripts showed moderate upregulation at all time points (FC = 2.06, 2.27, 1.90 for 24, 48, and 72 h, respectively). We also detected transcripts outside the top 20 that are associated with the immune response. Notably, defensin, another antimicrobial peptide, which was upregulated at all time points (FC = 1.75, 1.75, 1.65 for 24, 48, and 72 h, respectively).

Among the downregulated transcripts, several were also associated with cellular homeostasis (e.g., DNA/RNA polymerase superfamily, metazoan signal recognition particle RNA, receptor protein-tyrosine kinase, P-type ATPase; subfamily IV, P-type ATPase, occludin homology domain, cadherin-like/Cadherin conserved site and exostosin-like/exostosin-1) and immune function (e.g., toll-like receptor, receptor protein-tyrosine kinase) (Table 2). The most downregulated transcript, AALF003258-RA (FHA domain/G-patch domain/SMAD/FHA domain superfamily), plays a role in cellular homeostasis. Regarding immune-related transcripts, two downregulated genes were identified: AALF022033-RA and AALF011390-RA (Table 2). AALF022033-RA is linked to Toll/interleukin-1 receptor signaling and was downregulated at all time points (FC values between 2.52 and 2.78). At 72 h post infection, AALF011390-RA, encoding an ecdysone-induced protein, had a decreased expression equal to 2.57 FC. Additional transcripts not in the top 20 included leucine-rich immune proteins, which was downregulated at 48 h (FC = 1.96) and 72 h (FC = 1.58).

### Gene ontology analysis

**Fig. 5:**
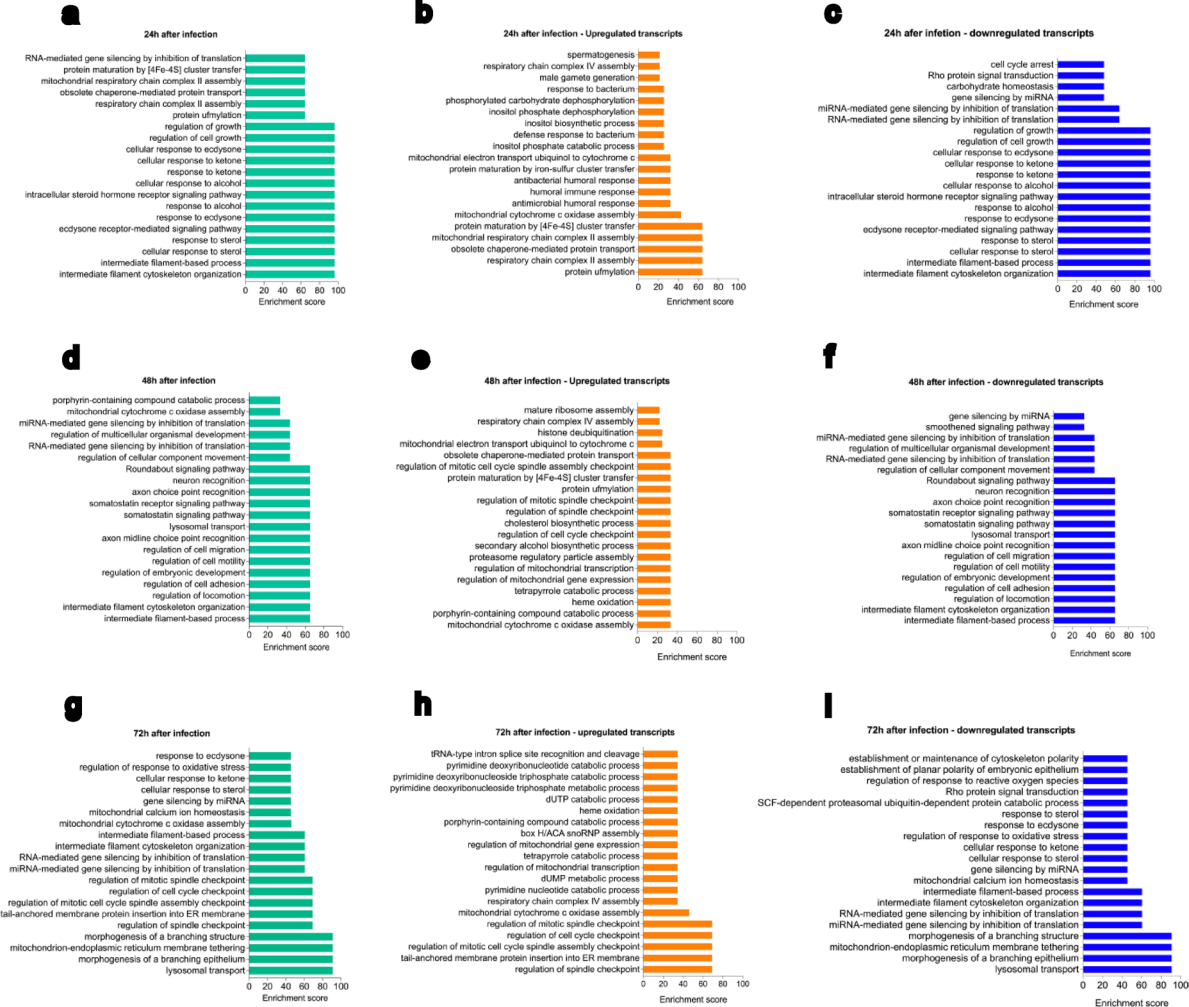
Gene Ontology (GO) enrichment analysis of biological processes in *Aedes albopictus* U4.4 cells following KRV infection. (**a**, **d**, **g**) Top 20 GO terms (ranked by enrichment fold values) for all differentially expressed transcripts (adjusted *p* ≤ 0.05; FC ≥ 1.5). (**b**, **e**, **h**) Top 20 GO terms for upregulated transcripts (adjusted *p* ≤ 0.05; FC ≥ 1.5). (**c**, **f**, **i**) Top 20 GO terms for downregulated transcripts (adjusted *p* ≤ 0.05; FC ≥ 1.5).

Gene Ontology (GO) analysis of DE transcripts (padj ≤ 0.05, FC ≥ 1.5) revealed enrichment of biological processes related to both cellular homeostasis and immune responses (Fig. 5a–i). At 24 hpi, several of the top 20 enriched GO terms (based on enrichment fold values) were associated with cellular homeostasis, including protein ufmylation and RNA-mediated gene silencing. Among immune-related categories, enrichment was observed for response to sterol and response to ketone (Fig. 5a). When stratified by expression direction, upregulated transcripts were enriched for immune-related terms such as defense response to bacterium and antimicrobial humoral response, while downregulated transcripts were linked to carbohydrate homeostasis (Fig. 5b–c). At 48 hpi, the top GO terms again included processes related to cellular homeostasis (e.g., RNA-mediated gene silencing, somatostatin receptor signaling pathway, roundabout signaling pathway) and immune function (e.g., regulation of cell adhesion, gene silencing by miRNA, lysosomal transport) (Fig. 5d). The stratification revealed enrichment of protein ufmylation among upregulated transcripts, while RNA-mediated gene silencing predominated among downregulated transcripts (Fig. 5e–f). At 72 hpi, the top 20 enriched GO terms remained consistent with earlier time points, comprising processes associated with cellular homeostasis (e.g., RNA-mediated gene silencing, regulation of response to oxidative stress) and immune response (e.g., response to ecdysone, response to ketone, response to sterol) (Fig. 5g). The stratification also showed that most GO terms associated with downregulated transcripts were linked to immune functions, including response to ecdysone, gene silencing by miRNA, miRNA-mediated inhibition of translation, and lysosomal transport (Fig. 5h–i).

## Discussion

The results confirm that, as have been shown also previously by Besson et al. (2024), the RNAi pathway in *Aedes albopictus* cells is activated following KRV infection. We show that the production of vsRNAs increased markedly between 24 and 72 hpi. The majority of these vsRNAs belonged to the vsiRNA population, with a pronounced enrichment toward the 3′ UTR of the KRV genome. This skewed distribution is consistent with the findings by Besson et al. (2024), who showed that classical insect-specific flaviviruses (cISF), and in particular KRV, exhibit a bias of vsiRNA production toward the 3′ UTR at 72 hpi^26^. Importantly, our data show that this 3′ UTR enrichment is already evident at earlier time points (24 and 48 hpi), indicating that this biased vsiRNA distribution is established early during infection. In contrast, mosquito-borne flaviviruses, such as West Nile virus, Zika virus and dengue virus generated vsiRNAs that were more uniformly distributed across the genome^26^. Together, these findings suggest that KRV induces a distinct strategy that results in a high generation of vsiRNA that targets the 3′ UTR, possibly to modulate the antiviral RNAi response independently of subgenomic flavivirus RNAs (sfRNAs) as suggested by Besson et al. (2024).

In addition to vsiRNAs, we observed the accumulation of longer small RNAs (25–30 nt) with features consistent with piRNA-like molecules. These piRNA-like reads displayed a non-uniform genomic distribution with distinct hotspots, particularly within coding regions corresponding to NS1, NS3, NS4A/B, and NS5, and showed increasing abundance over time. Notably, these reads exhibited a strong strand bias, with the majority mapping to the positive strand, while relatively few reads were detected on the opposite strand. Furthermore, nucleotide bias analysis revealed a strong enrichment for uridine at position 1 (1U; 73%) and adenine at position 10 (10A; 67%) in reads derived from the positive strand, whereas reads mapping to the negative strand showed no pronounced nucleotide preference (~ 25%). Together, these features are characteristic of piRNA-like processing and are consistent with a ping-pong-like amplification mechanism. An additional consideration is the potential contribution of endogenous viral elements (EVEs) to vpiRNA production, as integration of viral sequences into the mosquito genome has been shown to generate piRNAs in other systems^27^. While we cannot exclude this possibility, the strong temporal increase in virus-derived small RNAs following infection supports an origin primarily linked to active viral replication.

The contrasting mapping patterns of vsiRNAs and piRNA-like small RNAs, where vsiRNAs are enriched toward the 3′ UTR and piRNA-like reads form discrete hotspots within coding regions, suggest that these pathways may act on different regions or substrates of viral RNA and likely play complementary roles in the antiviral response. Specifically, vsiRNAs are likely derived from double-stranded RNA replication intermediates, whereas piRNA-like reads may originate from locus-specific processing of viral RNA fragments. This interpretation is supported by the observed strand asymmetry and nucleotide biases, which are hallmarks of piRNA pathway activity in insects.

While Besson et al. (2024) provided a detailed characterisation of KRV-derived small RNAs in U4.4 cells, the present study extends these findings by integrating small RNA profiling with transcriptomic analysis, thereby linking RNAi responses to broader host cellular and immune processes. Notably, despite the robust production of viral small RNAs, we did not observe strong transcriptional upregulation of canonical RNAi pathway genes, suggesting that RNAi activity may be regulated post-transcriptionally or constitutively expressed in these cells.

Transcriptomic analyses provided additional insights into the host cellular and immune responses to KRV infection. Although most fold changes were moderate, thousands of transcripts were differentially expressed across all three time points (24, 48 and 72 hpi). Among the upregulated transcripts, we identified genes associated with cellular homeostasis and immune or inflammatory responses (Table 1; Fig. 5b, e and h). Notably, the expression of glutathione transferase, protein p8, and defensin increased after KRV infection. Previous studies have shown that antioxidant genes such as glutathione peroxidase and glutathione S-transferase are upregulated in *Aedes albopictus* cells under xenobiotic stress, contributing to metabolic adaptation^28^. Similarly, glutathione S-transferase expression increases following dengue virus 2 infection, supporting intracellular conditions favorable for viral replication^29^. The transcription factor p8 is widely expressed during immune activation and has been shown to increase under inflammatory stress and during severe sepsis triggered by bacterial lipopolysaccharides^30^. The upregulation of defensin in *Aedes albopictus* cells upon KRV infection aligns with earlier reports in *Aedes aegypti* and *Aedes albopictus* exposed to xenobiotic or microbial stress^31,32^. While defensin is well known for its antimicrobial activity, its antiviral function is less clear. In one study, defensin produced in response to Japanese encephalitis virus exerted weak antiviral effects but paradoxically also enhanced viral adsorption and infectivity^33^. We also observed increased transcription of multiple cecropin isoforms, members of a major class of antimicrobial peptides in *Aedes* species^31^. Differential expression of cecropin transcripts has been reported following dengue virus 2 infection in *Aedes aegypti*^34^. In the same study, the authors also reported that other antimicrobial peptides, such as attacin and diptericin, were upregulated upon infection. In contrast, our results did not show significant changes in attacin or diptericin expression. Histone H3 Lys4 methyltransferase, known to regulate DNA repair, cell division, metabolism, and transcription in Drosophila^35^, was upregulated, underscoring the complex transcriptional reprogramming induced by KRV infection.

Among downregulated transcripts, several were linked to cellular homeostasis and immune signaling (Table 2; Fig. 5c, f and i). In particular, transcripts corresponding to Toll-like receptors were significantly reduced across all time points. The Toll pathway is a central immune signaling route in mosquitoes, initiating antimicrobial peptide production and cellular responses against invading pathogens^9^. In *Aedes aegypti*, Toll signaling mediates resistance to multiple dengue serotypes^34^. Therefore, Toll receptor downregulation may represent a KRV-driven mechanism to suppress host immunity and facilitate viral persistence. At 72 hpi, transcripts encoding a putative ecdysone-induced protein were significantly downregulated. Ecdysone, a steroid hormone, regulates the balance between immunity and reproduction in *Aedes aegypti*^36^. Suppression of ecdysone signaling has been linked to enhanced immune deficiency (Imd) pathway activation and greater pathogen resistance^37^. RNA interference-mediated depletion of ecdysone receptor inducing an increased expression of the immune deficiency pathway components, and mosquitoes became more resistant to infection by pathogens^37^. Hence, downregulation of ecdysone-induced genes may reflect viral modulation of hormonal signaling to alter host physiology. Although not among the top 20 most downregulated transcripts, we also observed reduced expression of a leucine-rich immune protein, previously shown to play antiviral roles against Zika and chikungunya viruses^38^. Its downregulation further supports a virus-driven suppression of immune effectors.

GO analysis revealed that multiple cellular pathways were affected during KRV infection (Figs. 5). Across all time points, enriched GO terms primarily corresponded to biological processes related to cellular homeostasis and immune function, with dynamic temporal shifts. Immune-related pathways were predominantly upregulated between 24 and 48 h, followed by downregulation at 72 hpi. Conversely, pathways associated with cellular homeostasis were mainly upregulated at 72 hpi. The early activation (24–48 h) of immune pathways likely reflects an active antiviral response, consistent with previous reports describing early upregulation of recognition and immune activation genes in insect-specific virus infections^21,24^. By 72 hpi, the observed downregulation of immune-related transcripts, including response to ecdysone, gene silencing by miRNA, and lysosomal transport, suggests a shift toward viral modulation of host defenses, supporting persistent infection.

It is important to note that this study was conducted in vitro using a mosquito-derived cell line, and caution should be exercised when extrapolating these findings to in vivo systems, where additional tissue-specific and systemic immune responses may influence infection dynamics.

In summary, this study provides new insights into the RNAi and transcriptomic responses of *Aedes albopictus* cells to KRV infection. By integrating small RNA and gene expression analyses, we show that KRV infection induces a coordinated host response characterised by a dominant siRNA pathway alongside the emergence of piRNA-like small RNAs with strand bias, nucleotide bias, and genomic hotspot patterns. In parallel, transcriptomic profiling revealed dynamic modulation of immune and cellular homeostasis pathways, including early activation of immune-related processes followed by their suppression at later stages, suggesting a transition toward a state permissive for persistent infection. Together, these features are consistent with a commensal-like virus–host interaction, in which viral replication is maintained without strong cytopathic effects while host responses are modulated rather than fully activated. These findings contribute to a broader understanding of insect-specific virus–host interactions in mosquito cells and provide a basis for future investigations into their potential relevance for vector competence and arbovirus control strategies.

## Material and methods

### Cell culture

*Aedes albopictus* U4.4 cells (kindly provided by Associate Professor G. Pijlman, Wageningen University, The Netherlands) and C6/36 cells (Sigma-Aldrich, Darmstadt, Germany) were maintained at 28 °C in Leibovitz’s L-15 medium (Gibco, Paisley, UK) supplemented with 10% fetal bovine serum (FBS; Gibco, Paisley, UK), 10% tryptose phosphate broth (TPB; Gibco, Paisley, UK), amphotericin B (250 µg mL^−1^; Gibco, Grand Island, NY, USA), and penicillin–streptomycin (100 U mL^−1^ penicillin + 100 µg mL^−1^ streptomycin; Gibco, Grand Island, NY, USA).

### Virus propagation and RNA quantification

The insect-specific flavivirus KRV (strain SR-75) was obtained from the European Virus Archive Global (EVAg). Virus stocks were propagated in C6/36 cells until a clear cytopathic effect (CPE) was observed (4 days post infection, dpi). Supernatants were harvested, centrifuged, and stored at − 80 °C.

Viral RNA levels were quantified by RT-qPCR. Plasmid standards containing the PCR target region of KRV (GenScript Biotech, The Netherlands) were used to generate standard curves. Results are reported as cDNA copy equivalents per µL. Because the plasmid standards do not account for reverse transcription efficiency, these values should be interpreted as relative estimates of viral abundance rather than absolute cDNA copy numbers. RNA extraction and RT-qPCR procedures are described below. RNA extraction and qRT-PCR procedures are described below.

### In vitro infection

U4.4 cells were seeded in 24-well plates and grown to 85–90% confluence (≈ 3.5 × 10^5^ cells per well). Cells were infected in triplicate for each time point using an inoculum corresponding to 0.1 RNA copies cell^−1^ in 200 µL infection medium (L-15 medium supplemented with 2% FBS and 10% TPB). After 1 h incubation at 28 °C, the inoculum was removed and replaced with 500 µL L-15 medium containing 5% FBS, 10% TPB, and antibiotics. Mock-infected cells served as controls.

Cells were sampled every 24 h up to 72 h post infection for RNA extraction, small RNA sequencing, and transcriptomic analyses. Supernatants were collected from 24 to 96 h p.i. for verification of viral replication by qPCR (data not shown).

### RNA extractions

RNA for growth curves and viral titration was extracted from 200 µL of supernatant or viral stock using 750 µL TRIzol reagent (Thermo Fisher Scientific, Carlsbad, CA, USA). After chloroform extraction and centrifugation, the aqueous phase was mixed 1:1 with 70% ethanol and purified using GeneJet spin columns (Thermo Fisher Scientific, Vilnius, Lithuania). RNA was eluted in 40 µL nuclease-free water and stored at − 80 °C.

For high-throughput sequencing, small (< 200 nt) and long (> 200 nt) RNAs were isolated from cells lysed directly in 750 µL TRIzol per well. RNA was extracted as above, and small and long RNA fractions were separated using the mirVana PARIS Kit (Thermo Fisher Scientific, Vilnius, Lithuania) according to the manufacturer’s instructions.

### Reverse transcription quantitative PCR

First-strand cDNA was synthesized using the SuperScript III Reverse Transcription Kit (Thermo Fisher Scientific, Carlsbad, CA, USA) with random hexamers and 5 µL RNA input per 20 µL reaction. Quantitative PCR was performed with iTaq Universal SYBR Green Supermix (Bio-Rad Laboratories, Hercules, CA, USA) using 2 µL cDNA and 0.5 µM of each primer in 20 µL total volume. Amplification was run on a Bio-Rad CFX96 system under the following conditions: 95 °C for 30 s, followed by 40 cycles of 95 °C for 7 s and 60 °C for 30 s, with plate reads after each cycle. Melt curves were generated from 60 °C to 96 °C at 0.5 °C increments.

Primers were designed with Primer3^39^ to yield 170–200 bp products (Tm = 60 °C). Reference genomes were retrieved from NCBI (Table 3).

**Table 3:** Primer pairs used for the qPCR analysis.

Primers	Binding site	Sequence (5′ ➔ 3′)	Ref. genome
KRV -F	5744–5763	TACGACCAATGGTGGACTCA	AY149904.1
KRV-R	5905–5924	ATTTGTGCCTCTGGCCAACA	

### High-throughput sequencing

Small and long RNA samples were quantified and quality-checked using an Agilent 4150 TapeStation with the RNA ScreenTape Kit (Agilent Technologies, Santa Clara, CA, USA).

For small RNA sequencing, triplicate samples from each time point were pooled and submitted to SciLifeLab (Stockholm, Sweden). Libraries (one per time point) were prepared using the QIAseq miRNA Low Input Kit (QIAGEN, Hilden, Germany) and sequenced on an Illumina NextSeq 2000 using a P2 flow cell with 101 nt (Read 1) + 8 nt (Index 1) setup, generating 10–20 million reads per sample (1 × 100 bp).

For long RNA sequencing, triplicate samples per time point were processed individually using the Illumina TruSeq Stranded mRNA (Poly-A selection) Kit and sequenced on an Illumina NovaSeq 6000 (S4 flow cell, 2 × 151 bp), producing ~ 30–40 million reads per sample.

The sequencing datasets from this study have been submitted to the NCBI Sequence Read Archive (SRA) (http://www.ncbi.nlm.nih.gov/sra/). The small RNA BioProject accession number is PRJNA870316 (sequence dataset accession numbers are SRX17115329—SRX17115334). For the long RNA sequencing sets the BioProject for the KRV infected cells is PRJNA141589 (sequence dataset accession numbers: SAMN54951829—SAMN54951837) and for the mock-infected cells PRJNA786637 (sequence dataset accession numbers: SRR17148808—SRR17148816).

### Small RNA sequence analysis

Raw reads (FASTQ files) were trimmed and filtered using Trim Galore! (v0.6.6), discarding reads < 17 nt or > 40 nt. Trimmed reads were mapped to the KRV genome (AY149905) with CLC genomic workbench (v26.0.1) using the parameter: Similarity fraction = 0.95. All reads were normalized by library size as reads per million (RPM) and the read length distributions were visualized in Prism 10. The SAM files from the mapping were further analyzed with MISIS-2 (v2.6)^40^ to visualize the alignment and polarity distribution of small RNA to the viral genomes. Ping-pong signatures of 25–30 nt piRNA were calculated and analyzed with WebLogo^41^.

### Transcriptomic data analysis

Long RNA (> 200 nt) data were analyzed using the nf-core/rnaseq pipeline (v3.12.0) implemented in Nextflow (https://nf-co.re/rnaseq/3.12.0)^42^. Steps included quality control (FastQC v0.11.9), adapter trimming (Trim Galore! v0.6.6), rRNA removal (SortMeRNA v4.2.0), and alignment with the STAR-Salmon workflow (Salmon v1.4.0). The *Aedes albopictus* Foshan genome (version Aalo1.2; https://vectorbase.org/vectorbase/app/downloads/Current_Release/AalbopictusFoshan/) served as reference. Default pipeline parameters were used unless otherwise stated. The workflow was executed on the Rackham high-performance computing cluster (UPPMAX, Uppsala University, Sweden; https://www.uppmax.uu.se/). Differential expression analysis was performed in RStudio using DESeq2, comparing infected versus mock-infected groups for each time point (24, 48, 72 hpi). Genes with low counts (sum of counts < 10 across all samples) were excluded prior to analysis. Wald tests were used to assess differential expression, and p-values were adjusted for multiple testing using the Benjamini–Hochberg method. Transcripts with adjusted *p* ≤ 0.05 were considered significantly differentially expressed, and those with fold change (FC) ≥ 1.5 were included in downstream analyses. Transcript identities were annotated using VectorBase (https://vectorbase.org/vectorbase/app/#transcriptomic-resources) (September 2025). Gene Ontology (GO) enrichment analyses were performed on the VectorBase platform, with emphasis on genes implicated in antiviral immune responses.

## Data Availability

The sequencing datasets from this study have been submitted to the NCBI Sequence Read Archive (SRA) (http://www.ncbi.nlm.nih.gov/sra/). The small RNA BioProject accession number is PRJNA870316 (sequence dataset accession numbers are SRX17115329—SRX17115334). For the large RNA sequencing sets the BioProject for the KRV infected cells is PRJNA141589 (sequence dataset accession numbers: SAMN54951829—SAMN54951837) and for the mock-infected cells PRJNA786637 (sequence dataset accession numbers: SRR17148808—SRR17148816).

## References

[1] David, S. & Abraham, A. M. Epidemiological and clinical aspects on West Nile virus, a globally emerging pathogen. *Infect Dis. (Lond)* **48**, 571–586. 10.3109/23744235.2016.1164890 (2016). 27207312 10.3109/23744235.2016.1164890

[2] Ferraris, P., Yssel, H. & Misse, D. Zika virus infection: An update. *Microbes Infect* **21**, 353–360. 10.1016/j.micinf.2019.04.005 (2019). 31158508 10.1016/j.micinf.2019.04.005

[3] Guo, C. et al. Global epidemiology of dengue Outbreaks in 1990–2015: A systematic review and meta-analysis. *Front Cell Infect Microbiol* **7**, 317. 10.3389/fcimb.2017.00317 (2017). 28748176 10.3389/fcimb.2017.00317 PMC5506197

[4] WHO. (World Health Organization, 2012)

[5] Luz, P. M., Vanni, T., Medlock, J., Paltiel, A. D. & Galvani, A. P. Dengue vector control strategies in an urban setting: An economic modelling assessment. *Lancet* **377**, 1673–1680. 10.1016/S0140-6736(11)60246-8 (2011). 21546076 10.1016/S0140-6736(11)60246-8 PMC3409589

[6] Sinkins, S. P. & Gould, F. Gene drive systems for insect disease vectors. *Nat. Rev. Genet.* **7**, 427–435. 10.1038/nrg1870 (2006). 16682981 10.1038/nrg1870

[7] Terenius, O., Marinotti, O., Sieglaff, D. & James, A. A. Molecular genetic manipulation of vector mosquitoes. *Cell Host Microbe* **4**, 417–423. 10.1016/j.chom.2008.09.002 (2008). 18996342 10.1016/j.chom.2008.09.002 PMC2656434

[8] Cheng, G., Liu, Y., Wang, P. & Xiao, X. Mosquito defense strategies against viral infection. *Trends Parasitol.* **32**, 177–186. 10.1016/j.pt.2015.09.009 (2016). 26626596 10.1016/j.pt.2015.09.009 PMC4767563

[9] Kumar, A. et al. Mosquito innate immunity. *Insects*10.3390/insets9030095 (2018). 30096752 10.3390/insects9030095 PMC6165528

[10] Prasad, A. N., Brackney, D. E. & Ebel, G. D. The role of innate immunity in conditioning mosquito susceptibility to West Nile virus. *Viruses* **5**, 3142–3170. 10.3390/v5123142 (2013). 24351797 10.3390/v5123142 PMC3967165

[11] Liu, Q. et al. The emerging role of STING in insect innate immune responses and pathogen evasion strategies. *Front. Immunol.* **13**, 874605. 10.3389/fimmu.2022.874605 (2022). 35619707 10.3389/fimmu.2022.874605 PMC9127187

[12] Patterson, E. I., Villinger, J., Muthoni, J. N., Dobel-Ober, L. & Hughes, G. L. Exploiting insect-specific viruses as a novel strategy to control vector-borne disease. *Curr. Opin. Insect Sci.* **39**, 50–56. 10.1016/j.cois.2020.02.005 (2020). 32278312 10.1016/j.cois.2020.02.005 PMC7302987

[13] Boehm, E. C. et al. *Wolbachia*-mediated resistance to Zika virus infection in *Aedes aegypti* is dominated by diverse transcriptional regulation and weak evolutionary pressures. *PLoS Negl. Trop. Dis.* **17**, e0011674. 10.1371/journal.pntd.0011674 (2023). 37782672 10.1371/journal.pntd.0011674 PMC10569609

[14] Rocha, M. N. et al. Pluripotency of Wolbachia against Arboviruses: the case of yellow fever. *Gates Open Res.* **3**, 161. 10.12688/gatesopenres.12903.2 (2019). 31259313 10.12688/gatesopenres.12903.2 PMC6561079

[15] She, L. et al. Wolbachia mediates crosstalk between miRNA and Toll pathways to enhance resistance to dengue virus in Aedes aegypti. *PLoS Pathog.* **20**, e1012296. 10.1371/journal.ppat.1012296 (2024). 38885278 10.1371/journal.ppat.1012296 PMC11213346

[16] Blitvich, B. J. & Firth, A. E. Insect-specific flaviviruses: a systematic review of their discovery, host range, mode of transmission, superinfection exclusion potential and genomic organization. *Viruses* **7**, 1927–1959. 10.3390/v7041927 (2015). 25866904 10.3390/v7041927 PMC4411683

[17] Goenaga, S. et al. Potential for co-infection of a mosquito-specific flavivirus, Nhumirim virus, to block West Nile virus transmission in mosquitoes. *Viruses* **7**, 5801–5812. 10.3390/v7112911 (2015). 26569286 10.3390/v7112911 PMC4664984

[18] Hall-Mendelin, S. et al. The insect-specific palm creek virus modulates west Nile virus infection in and transmission by australian mosquitoes. *Parasit Vectors* **9**, 414. 10.1186/s13071-016-1683-2 (2016). 27457250 10.1186/s13071-016-1683-2 PMC4960669

[19] Hobson-Peters, J. et al. A new insect-specific flavivirus from northern Australia suppresses replication of West Nile virus and Murray Valley encephalitis virus in co-infected mosquito cells. *PLoS ONE* **8**, e56534. 10.1371/journal.pone.0056534 (2013). 23460804 10.1371/journal.pone.0056534 PMC3584062

[20] Kenney, J. L., Solberg, O. D., Langevin, S. A. & Brault, A. C. Characterization of a novel insect-specific flavivirus from Brazil: Potential for inhibition of infection of arthropod cells with medically important flaviviruses. *J. Gen. Virol.* **95**, 2796–2808. 10.1099/vir.0.068031-0 (2014). 25146007 10.1099/vir.0.068031-0 PMC4582674

[21] Ohlund, P., Hayer, J., Hesson, J. C. & Blomstrom, A. L. Small RNA response to infection of the insect-specific lammi virus and hanko virus in an aedes albopictus cell line. *Viruses*10.3390/v13112181 (2021). 34834988 10.3390/v13112181 PMC8620693

[22] Ohlund, P., Lunden, H. & Blomstrom, A. L. Insect-specific virus evolution and potential effects on vector competence. *Virus Genes* **55**, 127–137. 10.1007/s11262-018-01629-9 (2019). 30632016 10.1007/s11262-018-01629-9 PMC6458977

[23] Huhtamo, E. et al. Characterization of a novel flavivirus from mosquitoes in Northern Europe that is related to mosquito-borne flaviviruses of the tropics. *J. Virol.* **83**, 9532–9540. 10.1128/JVI.00529-09 (2009). 19570865 10.1128/JVI.00529-09 PMC2738272

[24] Ohlund, P., Delhomme, N., Hayer, J., Hesson, J. C. & Blomstrom, A. L. Transcriptome analysis of an aedes albopictus cell line single- and dual-infected with Lammi Virus and WNV. *Int. J. Mol. Sci.*10.3390/ijms23020875 (2022). 35055061 10.3390/ijms23020875 PMC8777793

[25] Lutomiah, J. J., Mwandawiro, C., Magambo, J. & Sang, R. C. Infection and vertical transmission of Kamiti river virus in laboratory bred Aedes aegypti mosquitoes. *J. Insect Sci.* **7**, 1–7. 10.1673/031.007.5501 (2007). 20337552 10.1673/031.007.5501 PMC2999455

[26] Besson, B., Overheul, G. J., Wolfinger, M. T., Junglen, S. & van Rij, R. P. Pan-flavivirus analysis reveals sfRNA-independent, 3’ UTR-biased siRNA production from an insect-specific flavivirus. *J. Virol.* **98**, e0121524. 10.1128/jvi.01215-24 (2024). 39404457 10.1128/jvi.01215-24 PMC11575252

[27] Ter Horst, A. M., Nigg, J. C., Dekker, F. M. & Falk, B. W. Endogenous viral elements are widespread in arthropod genomes and commonly give rise to PIWI-interacting RNAs. *J. Virol.*10.1128/JVI.02124-18 (2019). 30567990 10.1128/JVI.02124-18 PMC6401445

[28] Hamzah, S. N. et al. In vivo glutathione S-transferases superfamily proteome analysis: An insight into aedes albopictus mosquitoes upon acute xenobiotic challenges. *Insects*10.3390/insects13111028 (2022). 36354852 10.3390/insects13111028 PMC9698486

[29] Cheng, C. C., Sofiyatun, E., Chen, W. J. & Wang, L. C. Life as a vector of dengue virus: The antioxidant strategy of mosquito cells to survive viral infection. *Antioxidants (Basel)*10.3390/antiox10030395 (2021). 33807863 10.3390/antiox10030395 PMC8000470

[30] Kallwellis, K., Grempler, R., Gunther, S., Path, G. & Walther, R. Tumor necrosis factor alpha induces the expression of the nuclear protein p8 via a novel NF kappaB binding site within the promoter. *Horm. Metab. Res.* **38**, 570–574. 10.1055/s-2006-950503 (2006). 16981138 10.1055/s-2006-950503

[31] Fallon, A. M. & Sun, D. Exploration of mosquito immunity using cells in culture. *Insect Biochem. Mol. Biol.* **31**, 263–278. 10.1016/s0965-1748(00)00146-6 (2001). 11167096 10.1016/s0965-1748(00)00146-6

[32] Gao, Y., Hernandez, V. P. & Fallon, A. M. Immunity proteins from mosquito cell lines include three defensin A isoforms from Aedes aegypti and a defensin D from Aedes albopictus. *Insect Mol. Biol.* **8**, 311–318. 10.1046/j.1365-2583.1999.83119.x (1999). 10469248 10.1046/j.1365-2583.1999.83119.x

[33] Liu, K. et al. Mosquito defensins enhance Japanese encephalitis virus infection by facilitating virus adsorption and entry within the mosquito. *J. Virol.*10.1128/JVI.01164-20 (2020). 32796073 10.1128/JVI.01164-20 PMC7565626

[34] Ramirez, J. L. & Dimopoulos, G. The Toll immune signaling pathway control conserved anti-dengue defenses across diverse Ae. aegypti strains and against multiple dengue virus serotypes. *Dev. Comp. Immunol.* **34**, 625–629. 10.1016/j.dci.2010.01.006 (2010). 20079370 10.1016/j.dci.2010.01.006 PMC2917001

[35] Poreba, E., Lesniewicz, K. & Durzynska, J. Histone-lysine N-methyltransferase 2 (KMT2) complexes - a new perspective. *Mutat. Res. Rev. Mutat. Res.* **790**, 108443. 10.1016/j.mrrev.2022.108443 (2022). 36154872 10.1016/j.mrrev.2022.108443

[36] Wang, X. et al. The ecdysone-induced protein 93 is a key factor regulating gonadotrophic cycles in the adult female mosquito *Aedes aegypti*. *Proc. Natl. Acad. Sci. U. S. A.*10.1073/pnas.2021910118 (2021). 33593917 10.1073/pnas.2021910118 PMC7923369

[37] Wang, M. et al. Ecdysone signaling mediates the trade-off between immunity and reproduction via suppression of amyloids in the mosquito *Aedes aegypti*. *PLoS Pathog.* **18**, e1010837. 10.1371/journal.ppat.1010837 (2022). 36137163 10.1371/journal.ppat.1010837 PMC9531809

[38] Zhao, L., Alto, B. W. & Shin, D. Transcriptional profile of *Aedes aegypti* leucine-rich repeat proteins in response to Zika and Chikungunya viruses. *Int. J. Mol. Sci.*10.3390/ijms20030615 (2019). 30708982 10.3390/ijms20030615 PMC6386990

[39] Rozen, S. & Skaletsky, H. Primer3 on the WWW for general users and for biologist programmers. *Methods Mol. Biol.* **132**, 365–386. 10.1385/1-59259-192-2:365 (2000). 10547847 10.1385/1-59259-192-2:365

[40] Seguin, J., Otten, P., Baerlocher, L., Farinelli, L. & Pooggin, M. M. MISIS-2: A bioinformatics tool for in-depth analysis of small RNAs and representation of consensus master genome in viral quasispecies. *J. Virol. Methods* **233**, 37–40. 10.1016/j.jviromet.2016.03.005 (2016). 26994965 10.1016/j.jviromet.2016.03.005

[41] Crooks, G. E., Hon, G., Chandonia, J. M. & Brenner, S. E. WebLogo: a sequence logo generator. *Genome Res.* **14**, 1188–1190. 10.1101/gr.849004 (2004). 15173120 10.1101/gr.849004 PMC419797

[42] Ewels, P. A. et al. The nf-core framework for community-curated bioinformatics pipelines. *Nat. Biotechnol.* **38**, 276–278. 10.1038/s41587-020-0439-x (2020). 32055031 10.1038/s41587-020-0439-x

